# Physical exercise augmented cognitive behaviour therapy for older adults with generalised anxiety disorder (PEXACOG): study protocol for a randomized controlled trial

**DOI:** 10.1186/s13063-019-3268-9

**Published:** 2019-03-18

**Authors:** Silje Haukenes Stavestrand, Kristine Sirevåg, Inger Hilde Nordhus, Trond Sjøbø, Trygve Bruun Endal, Hans M. Nordahl, Karsten Specht, Åsa Hammar, Anne Halmøy, Egil W. Martinsen, Eva Andersson, Helene Hjelmervik, Jan Mohlman, Julian F. Thayer, Anders Hovland

**Affiliations:** 1 Faculty of Psychology, University of Bergen, Box 7800, NO-5020 Bergen, Norway; 2 Solli DPS, Osvegen 15, NO-5228 Nesttun, Norway; 3 Faculty of Medicine, University of Oslo, Box 1078, Blindern, NO-0316 Oslo, Norway; 4 Department of Mental Health, Norwegian University of Science and Technology, Box 8905, NO-7491 Trondheim, Norway; 5 Faculty of Medicine, K.G. Jebsen Centre for Neuropsychiatric Disorders, University of Bergen, Box 7800, NO-5020 Bergen, Norway; 6 The Swedish School of Sport and Health Sciences, GIH, Box 5626, SE-114 86 Stockholm, Sweden; 7 Department of Psychology, William Paterson University, 300 Pompton Road, Wayne, NJ 07470 USA; 8 Department of Psychology, Ohio State University, 1835 Neil Avenue, Columbus, OH 43210 USA; 9 St.Olavs Hospital HF, Nidaros DPS, Box 3250, Sluppen, NO-7006 Trondheim, Norway; 10 Division of Mental Health and Addiction, Oslo University Hospital, Oslo, Norway; 11 Kronstad DPS/Division of Psychiatry, Haukeland University Hospital, Box 1400, NO-5021 Bergen, Norway

**Keywords:** Generalised anxiety disorder, GAD, Older adults, Physical exercise, Cognitive behavioural therapy, CBT, RCT, Brain-derived neurotrophic factor, Executive function

## Abstract

**Background:**

Generalised anxiety disorder (GAD) is a frequent and severe anxiety disorder among older adults. GAD increases the risk of developing other disorders such as depression and coronary heart disease. Older adults with GAD exhibit a poorer response to cognitive behaviour therapy (CBT) compared to younger patients with GAD. The normal age-related cognitive decline can be a contributor to reduced treatment efficacy. One strategy for improving treatment efficacy is to combine CBT with adjunctive interventions targeted at improving cognitive functions. Physical exercise is a viable intervention in this regard. Increased levels of brain-derived neurotrophic factor may mediate improvement in cognitive function. The present study aims to investigate the proposed effects and mechanisms related to concomitant physical exercise.

**Methods:**

The sample comprises 70 participants aged 60–75 years, who have GAD. Exclusion criteria comprise substance abuse and unstable medication; inability to participate in physical exercise; and conditions which precludes GAD as primary diagnosis. The interventions are individual treatment in the outpatient clinic at the local psychiatric hospital, with two experimental arms: (1) CBT + physical exercise and (2) CBT + telephone calls. The primary outcome measure is symptom reduction on the Penn State Worry Questionnaire. Other measures include questionnaires, clinical interviews, physiological, biological and neuropsychological tests. A subset of 40 participants will undergo magnetic resonance imaging (MRI). After inclusion, participants undergo baseline testing, and are subsequently randomized to a treatment condition. Participants attend five sessions of the add-on treatment in the pre-treatment phase, and move on to interim testing. After interim testing, participants attend 10 sessions of CBT in parallel with continued add-on treatment. Participants are tested post-intervention within 2 weeks of completing treatment, with follow-up testing 6 and 12 months later.

**Discussion:**

This study aims to develop better treatment for GAD in older adults. Enhancing treatment response will be valuable from both individual and societal perspectives, especially taking the aging of the general population into account.

**Trial registration:**

ClinicalTrials.gov, NCT02690441. Registered on 24 February 2016.

**Electronic supplementary material:**

The online version of this article (10.1186/s13063-019-3268-9) contains supplementary material, which is available to authorized users.

## Background

GAD is a potentially severe anxiety disorder characterised by profound and uncontrollable worry that ranges over a number of events and activities [1]. Patients often have bodily experiences of restlessness, fatigue and muscle tension. They often report mood changes such as irritability and difficulty concentrating, in addition to sleep disturbance. While GAD is a severe and disabling condition in itself, it is also associated with an increased risk of comorbid depression [2]. Furthermore, anxiety disorders in general are associated with social disability, social isolation, physical inactivity and reduced quality of life [3–6]. GAD is highly prevalent in primary care settings, and is present in 22% of patients seeking help for anxiety in this context [7].

The estimates of lifetime prevalence of GAD range from 2.8 to 6.2% [8]. The estimates of prevalence of GAD in older adults range from 3.2% [9] to 11.5% [10]. In the replication of the National comorbidity study (NCS-R) by Kessler et al. [11, 12], GAD was estimated to affect 3.6% of adults above 60 years of age. Byers et al. [9] identified a 12-month 3.2% prevalence of GAD in older adults aged 55–64 years. This differs from the results in the longitudinal LASA study, where GAD was found to be the most prevalent anxiety disorder among older adults, with rates (6-month prevalence) ranging from 6.9% in the age group 75–85 years to 11.5% in those aged 65–74 years [10]. The discrepancies between studies in terms of prevalence could be influenced by the diagnostic system used. Beekman et al. [10] used criteria from the *Diagnostic and statistical manual of mental disorders* (DSM)-III, while the other studies have used DSM-IV criteria [9, 11, 12]. However, Zhang et al. [13] recently investigated the development of GAD in adults above 65 years of age, and found that more than 8.4% of this cohort developed GAD across a period of 12 years, in whom 80.0% of cases were of first onset.

Older adults appear to be particularly vulnerable to developing GAD and a peak in prevalence has been demonstrated in people aged 50 years and above [14, 15]. The causes of this peak are so far poorly understood. According to Chou et al. [15], further research is needed to adress this issue: they point out the presence of hypertension, poor role functioning, bodily pain, poor self-rated health status, lack of vitality, poor social functioning and poor role-emotional functioning in patients with late-onset GAD. These are, however, only factors associated with GAD and it is not known if treatment of GAD will improve these factors or if these factors contribute to the development in GAD in patients above 50 years of age. LeRoux and colleagues [14] did not find that experience with negative life events such as widowhood, poor health or cognitive impairment was related to the development of GAD in older adults. They do however discuss the insensitivity of their measures in terms of negative life events. Furthermore, they suggest that disability might be a risk factor for developing GAD in late life.

Cognitive behaviour therapy (CBT) is the recommended treatment for GAD [16]. CBT for GAD includes psychoeducation, progressive muscle relaxation, cognitive restructuring and graded exposure [17]. Even though CBT has generally been found to be more effective as a treatment in patients with GAD than in control conditions [18], older adults with GAD are less responsive to treatment than working-age adults [19, 20]. A recent meta-analysis identified lower effect sizes in older compared to working-age adults with GAD [21]. Several studies have attempted to improve the treatment efficacy in older adults with GAD, with promising results.

Wetherell and colleagues investigated the effects of combined antidepressant medication (escitalopram) with CBT, compared to escitalopram alone [22]. The participants first received acute treatment with escitalopram, followed by 16 weeks of either escitalopram alone or escitalopram + CBT, and a maintenance phase of 28 weeks with continued escitalopram. The CBT consisted of 16 sessions, and trained therapists delivered the CBT according to a manual. The combined CBT and escitalopram group had a significantly lower score on the Penn State Worry Questionnaire (PSWQ) following maintenance treatment, as compared to the escitalopram group alone [22]. The PSWQ is a self-report measure of worry severity, and a larger reduction in scores indicates a larger change in the respondent’s experience of worry severity [23]. The authors conclude that CBT can in fact augment the effect of medical treatment for GAD, and that both escitalopram and CBT can provide relapse prevention benefits. However, they also point out the limitations of the small sample size and lack of long-term follow up after the maintenance treatment phase.

Several authors suggest that the age-related cognitive decline, and especially a reduction in executive functions, could be an important mediator of the poorer treatment efficacy of CBT in older adults [24–26]. The cognitive functions most often affected by normal aging reflect executive performance, such as the ability to organize and plan activities, shift and direct attention and adjust behaviour according to long-term goals [27]. Consequently, some studies on augmenting the treatment of GAD in older adults have focused on add-on strategies designed to improve cognitive functions. Mohlman and colleagues published an article in 2003 on two pilot studies investigating the effects of standard and enhanced CBT for treatment of GAD in older adults, as compared to a wait-list condition [28]. Both the standard and enhanced CBT were based on the same treatment manual (13 weekly individual 50-min sessions). The enhanced CBT, however, additionally included learning and memory aids, which aimed to increase homework compliance, strengthen the memory of skills learned in treatment and facilitate the use of these skills. Participants receiving enhanced CBT were more homework-compliant than participants receiving standard CBT, and the treatment effect sizes were somewhat larger with enhanced than with standard CBT. The authors note that improvements in both groups were modest compared to effect sizes seen in younger samples, and suggest further investigations of the effectiveness of add-on strategies with CBT in older adults with GAD [28].

Mohlman and Gorman [24] conducted a pilot study whereby 32 adults aged 60 years or older received 13 individual weekly CBT sessions according to a published protocol, and 6-monthly booster sessions for 6 months consecutively. Participants underwent neuropsychological testing to examine executive function, and the authors initially categorized participants with either intact executive function, or executive dysfunction. However, a subset of the participants with executive dysfunction had improvements in executive functioning at the post-treatment neuropsychological assessment, and the authors thus categorized them as having improved executive function. The participants with intact and improved executive function improved the most in the treatment outcome measures post treatment and at follow up, whereas the participants with executive dysfunction neither improved in executive function nor in the outcome measures. The authors conclude that baseline executive dysfunction is not a clear predictor of treatment outcome in their study. However, their findings also implicate that in a subset of participants, executive dysfunction is associated with non-response to CBT [24].

One small pilot study has investigated whether an executive skills rehabilitation programme could augment the treatment efficacy of CBT in older adults with GAD [25]. Eight older adults received either CBT (13 weekly individual 50-min sessions) or CBT combined with executive skills training. The participants in the CBT + executive skill training condition had significantly better improvement on the PSWQ than the participants receiving standard CBT. The small sample size clearly limits the generalisability of the results, but points to targeting executive functions as a viable add-on strategy to improve the outcome of CBT in older adults with GAD.

In summary, the pilot studies on add-on strategies focusing on improving cognitive function all suggest that add-on strategies targeting age-related cognitive decline can be a viable approach in improving treatment efficacy in older adults with GAD, and recommend future research to systematically further examine the use of add-on interventions adjunct to CBT. Another suggested approach aimed at improving executive function is to include physical exercise as a part of the treatment programme [29].

Engaging in physical exercise over time has several demonstrated benefits, such as improved cognitive function and increased brain volume in the prefrontal and temporal cortices [30, 31] and preservation of hippocampal volume in older adults [32]. Physical fitness level is positively related to cognitive function, especially executive function, and to preservation of brain tissue [33, 34]. It has further been shown that aerobic exercise can increase the plasticity of the brain and this increase is associated with improved cognitive function [35]. A mechanism involved in the increased plasticity is the neurotrophin brain-derived neurotropic factor (BDNF) [36]. Improved cognitive function has been shown to be mediated by increased BDNF [37, 38]. BDNF protects the brain against the harmful effects of stress through support of neuronal plasticity, synaptogenesis, synaptic efficacy and neuronal survival [39]. BDNF decreases as a function of stress and is reduced in both anxious and depressed patients [40, 41], and increased in both healthy, depressed and anxious patients after physical exercise [40, 42]. Furthermore, BDNF levels relate to the effects of exposure-based interventions. The level of BDNF in the brain has been related to successful fear extinction [43], and peripheral BDNF has been shown to be positively related to the effectiveness of exposure-based treatment for post-traumatic stress disorder (PTSD) [44] and panic disorder [45].

Physical exercise is known to positively affect not only cognitive functions, but also cardiac regulation [46, 47], including heart rate variability (HRV). HRV is a predictor of all-cause mortality and death following myocardial infarction [48, 49]. Patients with anxiety disorders have reduced HRV [50], which is considered to result from altered inhibitory activity in the prefrontal cortex (PFC) [51]. Executive function and HRV have been found to be related both in healthy adults [52, 53] and in patients with anxiety [54]. Though HRV decreases with increasing age [55], it was recently demonstrated that physical exercise could increase HRV in healthy older adults [56] and that changes in HRV are related to the performance of physical exercise.

Previous studies investigating long-term physical exercise as an adjunct to with CBT for treatment of anxiety disorders have shown mixed results. One study had null findings [57], two studies indicate benefits of adjunct physical exercise [58, 59] and one feasibility study concluded that add-on physical exercise is feasible and potentially beneficial [60]. The study that did not find positive effects of CBT combined with physical exercise was in a non-clinical sample [57], which suggest a limited added effect of physical exercise in a non-clinical population. A recent study on physical exercise combined with exposure therapy for panic disorder demonstrated the lack of a significant effect of adjunct physical exercise. The authors point to ceiling effects as the reason for the lack of effect of the add-on treatment, as exposure therapy already has high treatment efficacy [61]. This is in line with a point made by other authors, that physical exercise could be a more potent intervention in groups in whom treatment with CBT has limited effects (e.g. [62]).

Based on this rationale, the aim of the present study is to compare the effects of physical-exercise-augmented CBT to standard CBT combined with a placebo control condition. We expect that exercise-augmented CBT will yield better results than standard CBT as measured by our primary outcome measure, symptom reduction on the Penn State Worry Questionnaire (PSWQ) [23]. The proportion of patients who achieve remission as assessed by an independent clinical assessor using the Anxiety Disorders Interview Schedule for DSM-IV (ADIS-IV) [63], will be assessed as a secondary outcome measure. We also expect levels of BDNF at baseline to be positively related to treatment outcome, and that the participants that receive augmented CBT will improve significantly in the cognitive measures and in levels of BDNF following treatment as compared to the CBT alone group. Furthermore, we expect HRV to improve with exercise-augmented CBT, and that these changes will correlate with clinical change, and changes in cognitive functions and BDNF levels.

## Method

### Study design

The study is a 2 × 2 mixed factorial design (Time x Group) with participants randomized to one of two treatment conditions: (1) CBT combined with physical exercise (CBT + PE) or (2) CBT combined with telephone call attention placebo (CBT + AP). Participants complete pre-treatment, interim and post-treatment outcome measures, in addition to follow-up tests at 6 and 12 months after treatment.

Participants in both treatment conditions attend a 5-week pre-treatment period. The pre-treatment period has a two-part rationale. The first 5 weeks of physical exercise are aimed at introducing the participant to the exercises with a focus on adjustment and neuromuscular facilitation [64]. In addition, resting BDNF has been shown to increase after 5 weeks of physical exercise [65]. The primary goal of the pre-treatment period is to ensure good implementation of physical exercise, and to obtain elevated resting BDNF in the CBT + PE group before the onset of CBT. As such, the pre-treatment period is a measure to facilitate the add-on effects of physical exercise.

Participants receiving CBT + PE attend one supervised and two unsupervised sessions of PE during each of the 5 weeks of the pre-treatment period. Participants receiving CBT + AP receive one telephone call per week during the pre-treatment period.

**Fig. 1:**
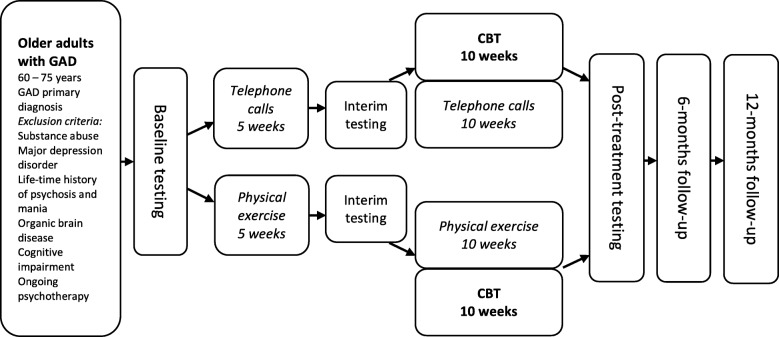
Study design. Visual presentation of the study design, including sample, assessments, and interventions. GAD, generalised anxiety disorder; CBT, cognitive behaviour therapy

**Fig. 2:**
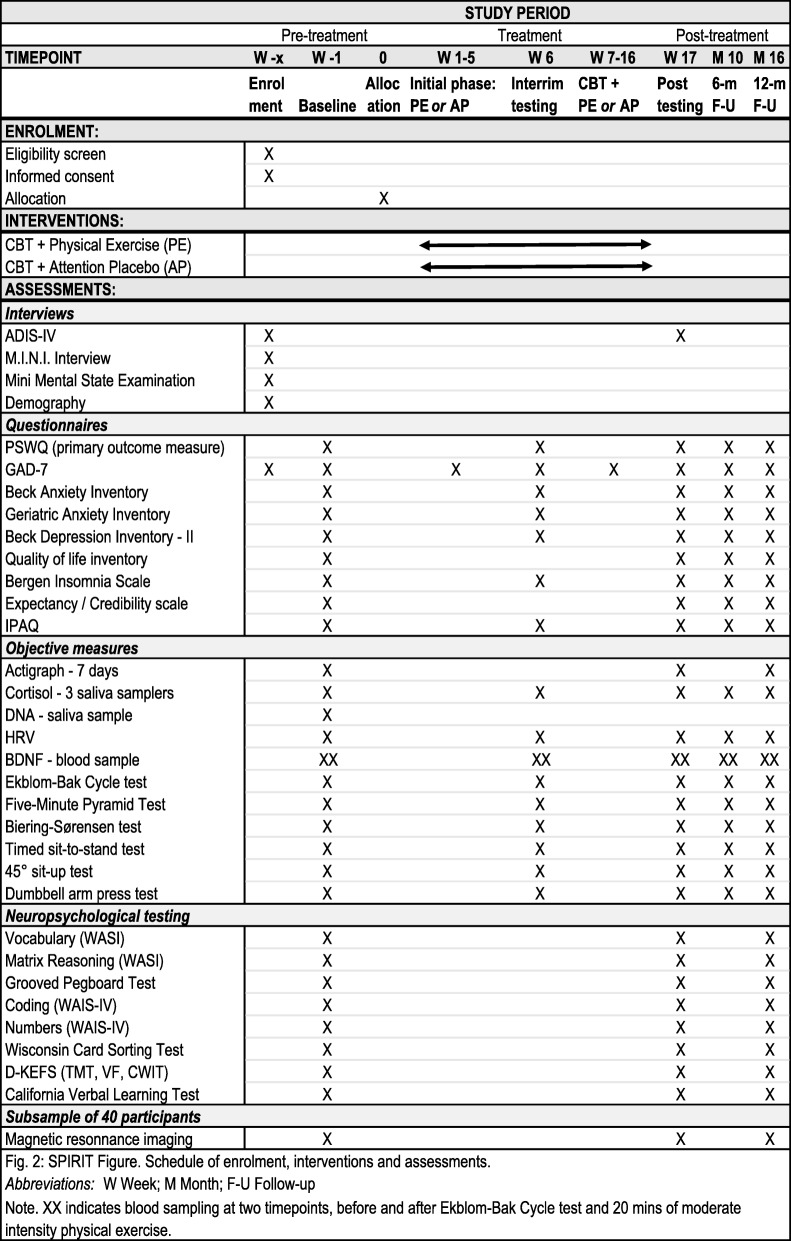
Standard protocol items: recommendation for interventional trials (SPIRIT) figure. Schedule of enrolment, interventions and assessments. Overview of the measures applied in the study. CBT, cognitive behaviour therapy; ADIS-IV, Anxiety Disorders Interview Schedule for DSM-IV; M.I.N.I., Mini International Neuropsychiatric Interview; PSWQ, Penn State Worry Questionnaire; GAD-7, Generalized Anxiety Disorder 7-item scale; IPAQ, International Physical Activity Questionnaire; HRV, heart rate variability; BDNF, brain-derived neurotropic factor; WASI, Wechsler Abbreviated Scale of Intelligence; WAIS-IV, Wechsler Adult Intelligence Scale – Fourth Edition; D-KEFS, Delis–Kaplan Executive Function System; TMT, Trail-Making Test; VF, Verbal Fluency; CWIT, Color Word Interference Test

After the pre-treatment period, the participants receive 10 weekly sessions of CBT combined with either continued physical exercise in the participants receiving CBT + PE or weekly telephone contact in parallel to the CBT sessions in the participants receiving CBT + AP. See Fig. 1 for a display of the study design. The present protocol has been prepared in accordance with relevant items from the Standard Protocol Items: Recommentations for Interventional Trials (SPIRIT) Checklist (see Additional file 1). See Fig. 2 for the SPIRIT figure displaying the schedule of enrolment, interventions and assessments.

### Participants

Adults, aged 60–75 years (*n* = 70), who have a primary diagnosis of GAD as evaluated by the ADIS-IV [63] will be included. Exclusion criteria are (1) substance abuse; (2) use of benzodiazepines and antipsychotic medication; (3) changes in the dose of other psychotropic medication during the study; (4) medical conditions that preclude participation in physical exercise; (5) severe major depression as determined by the Mini International Neuropsychiatric Interview (M.I.N.I.) [66, 67]; (6) life-time history of psychosis and/or mania; (7) participation in other ongoing psychotherapy; (8) organic brain disease; (9) a score of 25 or less on the Mini Mental State Examination (MMS-E) [68] and (10) participation in physical exercise exceeding 75% of the exercise dose given in the study.

A subsample of 40 participants, who do not the meet exclusion criteria for magnetic resonance imaging (MRI), will be included in a nested MRI study. The exclusion criteria for MRI are (1) MRI-incompatible metal screws or implants; (2) large tattoos on head, neck or upper body; (3) pacemaker and (4) claustrophobic fear, to a degree whereby participants refuse to undergo MRI without sedatives. These exclusion criteria are applied to ensure patient safety.

### Measures

#### Clinical interviews

During the diagnostic work before inclusion, we use the M.I.N.I. [66, 67] to determine comorbid psychiatric conditions and the ADIS-IV [63] to determine the presence of GAD. After treatment, an independent assessor determines remission from GAD using the ADIS-IV [63].

#### Questionnaires

The PSWQ [23] assesses the severity of symptoms in GAD. This is a 16-item self-report questionnaire whereby participants rate each item on a scale from 1 (not at all typical of me) to 5 (very typical of me). A higher score indicates a high level of worry. Five items are reverse-scored. The Beck Anxiety Inventory [69] assesses general anxiety. This is a 21-item self-report questionnaire whereby participants rate each item on a 0–3 scale, where 0 is “Not at all” and 3 is “Severely – it bothered me a lot”. The Beck Depression Inventory – II [70] measures the level of depression. This is a 21-item self-report questionnaire whereby each item is scored on a 0–3 scale. A higher score indicates greater symptom severity. The Generalized Anxiety Disorder Questionnaire [71] is a screening tool for GAD, and will be used as a session-by-session assessment and at each data collection point. This is a 7-item self-report questionnaire whereby participants rate themselves on a scale from 0 (not at all sure) to 3 (nearly every day). A higher score indicates greater symptom severity. The Geriatric Anxiety Inventory [72] measures general anxiety in older adults. This is a 20-item self-report questionnaire with “Agree/Disagree” categories. Agree is scored 1 and disagree 0, and a higher score indicates greater anxiety. Symptoms of insomnia will be assessed by the Bergen Insomnia Scale [73]. This is a 6-item self-report questionnaire whereby participants rate themselves on a scale from 0 to 7, indicating the number of days they report the problem. A higher score indicates more symptoms of insomnia. The Quality of Life Inventory [74] will be used to assess quality of life. This is a 32-item self-report questionnaire assessing diverse aspects of quality of life, such as “Health”, “Economy”, “Creativity” and “Love”. Each category is evaluated both by how important it is to the participant (from 1 (not important) to 3 (very important)) and by how satisfied they are with it (from − 3 (very dissatisfied) to 3 (very satisfied)). The Credibility/Expectancy Questionnaire [54] measures treatment expectancy. This is a 5-item self-report questionnaire whereby participants rate statements about treatment on a scale from 1 to 10, where a higher score indicates greater credibility. In addition, participants complete the International Physical Activity Questionnaire (IPAQ-short) [75, 76] to assess physical activity level. This is an 8-item self-report questionnaire, whereby the participant indicates how much time he or she has spent on high and moderate intensity physical activity, walking and sitting during the last 7 days.

We expect participants to spend 45–60 min completing the questionnaires at each test point. To avoid missing data, the participants complete the questionnaires with one of the project coordinators present. As such, participants can ask questions about the questionnaires, and the project coordinator can ensure that the participant has answered all questions.

#### Neuropsychological testing

IQ level will be assessed with two subtests, Vocabulary and Matrix Reasoning, from the Wechsler Abbreviated Scale of Intelligence [77]. Psychomotor speed and hand-eye coordination will be measured using the Grooved Pegboard Test [78], and the subtest, Coding, from the Wechsler Adult Intelligence Scale – Fourth Edition [79]. Executive functions will be assessed using the Wisconsin Card Sorting Test: Computer Version 4 research edition [80], alongside three subtests from Delis-Kaplan Executive Function System battery [81]: Trail Making Test, the Verbal Fluency Test, and the Color-Word Interference Test, and working memory is assessed using the subtest, Numbers, from the WAIS-IV [79]. Verbal memory will be measured using the California Verbal Learning Test [82].

#### Physiological and biological measures

Serum BDNF is measured in blood samples and assayed using the Human proBDNF and mBDNF DuoSets [83, 84]. We use an ambulatory electrocardiogram (ECG) [85] to measure HRV. Physical fitness is measured by the Ekblom-Bak submaximal cycle ergometer test [86], the Five-Minute Pyramid Test [87] is used for prediction of maximal oxygen uptake (VO_2_ max) and items from the Senior Fitness Test are used for measuring submaximal physical strength [88].

The study will investigate potential mediators and moderators of treatment outcome. Cortisol is measured in three saliva samples collected during the assessment days, and is analysed using the cortisol saliva luminescence immunoassay [89]. Genetic material is obtained by one saliva sample at baseline, and analysed using the TaqMan method [90]. Participants wear the Philips Actiwatch 2 [91] for 1 week at baseline, post-intervention and at 12-month follow up to measure activity level and sleep variables.

A subsample of 40 participants undergo MRI at baseline, post testing and at 12-month follow-up testing. MRI will be performed using a GE Signa 3 .0T twin speed HDxt scanner [92]. A head coil with video goggles allows for the Eriksen flanker task [93] to be applied during functional MRI.

### Procedures

This study will be conducted at an outpatient clinic at Solli DPS, a local mental health facility. The neuropsychological testing and MRI will take place at Haukeland University Hospital, in cooperation with the University of Bergen. There are five test points for each participant: baseline, interim, post-treatment and 6-month and 12-month follow up. We have designed the pattern of test points for the investigation both of treatment mechanisms and treatment outcome.

#### Baseline testing

The baseline testing consists of several elements: (1) a start-up conversation with a project coordinator, whereby the participant completes questionnaires, receives saliva samplers with instructions and receives an actigraph watch; (2) a physical/physiological test day, when the participants undergo ECG, provide a saliva sample for DNA testing and blood samples for measurement of BDNF and undergo tests of physical fitness and strength; (3) an appointment for neuropsychological testing and (4) an appointment for functional magnetic resonance imaging (fMRI).

#### Randomisation

Randomisation is performed anonymously at the University of Bergen in blocks of ten following baseline testing. Randomisation is computerised and concealed [94]. The person conducting the randomisation is blind to participants’ identities and does not have any information on the participants. Blocks of ten were preferred, to achieve predictability for the hospital and staff. This does, however, mean that for every block, the tenth allocation can be determined with certainty if that of the former nine is tracked. Calculations using hypergeometric distributions also show that there is a 4.76%, 16.66% and 44.44% probability that the precise allocation of participants can be known with certainty following 6, 7 and 8 draws respectively. However, information about allocation is not tracked, and was deemed an acceptable risk to achieve practical feasibility of running the trial within a clinical setting.

#### Blinding

This study is single-blinded. We do not disclose to the participants which of the adjunct interventions we expect to be the active intervention. Patients are informed that we investigate two adjunct strategies, and that we will evaluate which of these provides better treatment efficacy. We take great care to inform all involved personnel to convey this message.

The independent clinical assessor, who evaluates remission after treatment, does not know which adjunct intervention the participant received during treatment. The appointment with the independent clinical assessor is scheduled without giving the assessor access to the patient’s medical journal. Also, the project coordinator meets with each patient before the appointment and give instructions not to reveal treatment allocation. After the interview, we ask the independent clinical assessor whether the patient revealed treatment allocation, and make a record of the response.

#### Pre-treatment period

After randomization, the included participants go on to the pre-treatment period with either five weekly sessions of physical exercise or telephone call attention placebo. The pre-treatment period lasts 5 weeks, but can be extended by 2 weeks due e.g. to illness or holidays. If after the maximum extension period the patient has completed fewer than five sessions, the patient proceeds to interim testing. Compliance will be registered for each participant.

#### Interim testing

The interim testing consists of two appointments; a physical/physiological test day, in which participants undergo ECG, provide blood samples for measurement of BDNF, test physical fitness and strength, and are provided with take-home saliva samplers; and an appointment with a project coordinator, whereby the participant completes questionnaires and returns the saliva samplers.

#### Treatment

After interim testing, participants have a start-up conversation with their CBT therapist, followed by 10 weekly sessions of CBT combined with either physical exercise or telephone call attention placebo. The treatment period lasts approximately 11 weeks, but can be extended by 4 weeks due e.g. to illness or holidays. Interventions are scheduled on separate days of the week, so that the participant does not attend both CBT and supervised physical exercise on the same day. We register compliance for each participant.

#### Post-treatment testing

Post-treatment testing starts as soon as possible after completion of treatment, and no later than 2 weeks after the last treatment session. The post-treatment testing corresponds to the baseline testing, with the exception of the DNA saliva sample, which is only provided at baseline.

#### Follow-up testing at 6 months

The 6-month testing corresponds to the interim testing.

#### Follow-up testing at 12 months

The 12-month testing corresponds to the post-treatment testing.

### Interventions

#### Cognitive behaviour therapy (CBT)

Participants attend 10 sessions of individual CBT applied following a manual, in accordance with the protocol developed by Borkovec [17]. One initial session focuses on developing a therapeutic alliance and introducing the therapy before the CBT is applied. The duration of the CBT is approximately 10 weeks. The therapist and participant can agree to increase the number of weeks within reasonable limits, or do more than one session within one week, in case a participant misses one or more CBT sessions (e.g. because of holidays, illness, etc.). If the duration of the therapy exceeds 15 weeks, the participant will not receive the remaining sessions. The project coordinators keep a record of the number of CBT sessions each participant attends. We have adapted the CBT manual to the study in collaboration with Professor Hans M. Nordahl from the Norwegian University of Science and Technology. Professor Nordahl also provides training and supervision to the therapists who perform CBT, who are psychologists or psychiatrists with formal training in CBT and specific education in the study CBT manual. Before treating participants in the PEXACOG study, therapists are required to have performed one full video-documented therapy that has been rated by an external assessor and scored on the Cognitive Therapy Adherence and Competence Scale (CTACS) [95] with a mean score of 3 or higher. Two qualified assessors will evaluate at least 20% of the individual therapists’ treatment courses according to the following procedure: Therapists record all CBT sessions. The recorded sessions will be drawn randomly, stratified by first or second half of the treatment course (i.e. session 1–5 or 6–10). Both assessors will rate each selected recording on the covered domains in the CTACS, and CTACS ratings are compared to determine inter-rater reliability.

The CBT consists of several elements. Participants receive psychoeducation about anxiety and the treatment model. They also learn diaphragmatic breathing, and two versions of a progressive muscle relaxation exercise - one longer version with 7 muscle groups, and one shorter version with 4 muscle groups. These elements receive attention throughout the treatment. Participants complete homework assignments between sessions, including daily registration of anxiety level. Participants receive an mp3 player with recorded instructions on diaphragmatic breathing and progressive muscle relaxation to use between sessions. Furthermore, participants learn cognitive monitoring and restructuring. A key element of the CBT is self-controlled desensitisation. Self-controlled desensitisation is a process whereby participants establish an anxiety hierarchy, learn new responses to anxiety cues, and connect the anxiety cues with the new response. To practice self-controlled desensitisation, participants are asked to worry on purpose, and respond with relaxation when the anxiety levels are elevated. The final treatment session focuses on relapse prevention.

#### Physical exercise (PE)

The duration of the PE programme is 15 weeks. The first 5 weeks comprise the pre-treatment intervention. During the following 10 weeks the participants attend to both PE and CBT. Each week consist of one supervised training session at the local mental health facility (clinic), and two individual training sessions. Supervised sessions are led by physiotherapists or occupational therapist who are trained in the study manual and receive supervision on test procedures and the exercise manual. For the individual training sessions, each participant is given their own exercise journal with instructions, and is instructed to log each individual training session. All participants use a heart rate monitor watch [96], which is used during individual endurance training sessions and monitored by the PE therapist at the supervised training session. This is a measure of patient adherence to the PE. Physical tests at the interim and post testing time points also serve as manipulation checks. The participants complete the GAD-7 questionnaire at each of the supervised training sessions.

The PE programme is adjustable according to the participants’ prerequisites, without compromising the exercise intensity. Each session lasts approximately 45–60 min, and contains both strength and endurance training. The strength training programme consists of seven exercises; squats, push-ups, lunges, rowing with elastic bands, calf raises, dips and shrugs with elastic bands. Endurance training is designed based on interval principles. The exercise intensity progresses every fifth week. The intensity level at the beginning of the intervention period (week 1–5) will be low to moderate (65–85% of maximum heart rate (HR max)). It increases to moderate intensity at week 6–10 (65–90% of HRmax) and at week 11–15 it increases to moderate to high intensity (65–95% of HRmax). This equals 60–70%, 70–80% and 80–90% of one repetition maximum (1RM) [97] when transferred to resistance load in strength exercises [98]. The exercise programme is designed to ensure that participants take part in at least the minimal therapeutic dose of exercise, which is 150 min of moderate intensity activity per week [99, 100].

#### Telephone call attention placebo

The telephone call attention placebo lasts 15 weeks, with one telephone call scheduled each week. PhD candidates, who are trained clinical psychologists, conduct the telephone calls. The telephone contact has no treatment intention, and will follow an interview guide with the questionnaire GAD-7 and talking points such as “How was your week?” and “Do you have any plans for the week-end?”. Each call lasts no longer than 20 min. The intention of the telephone call attention placebo is to control for the extra therapist contact in the physical exercise condition, and to ensure that participants spend approximately the same net amount of time in direct therapist contact across the treatments. The first 5 weeks of telephone call attention placebo is an initial phase of treatment. During the following 10 weeks, telephone calls are combined with CBT.

### Recruitment and eligibility

We will recruit participants through primary health care services and the media. Assessment for the trial requires a formal referral from a primary physician to the hospital. The project coordinators apply the following interviews with potentially eligible participants: the M.I.N.I. [66, 67], the GAD-module from ADIS-IV [63] and the MMS-E [68]. If eligible for inclusion, participants sign an informed consent form, and complete self-report baseline measures. Furthermore, the project coordinators obtain written approval for participation from the participants’ primary physician to ensure their safety in participating in physical testing and exercise. The project coordinators schedule baseline testing once the primary physician has medically cleared the participant.

### Data management and security

Each potential participant is assigned a unique identification number at the start of the assessment. Only the project coordinators have access to the connecting key, which we store at a separate domain on a secure research server. We handle all data according to internal procedures, and store data on the hospital regional secure research server. We store biological material in a research-specific biobank in accordance with the Regional Committee for Medical and Health Research Ethics procedures. We will anonymize all data 5 years after trial completion.

### Ethical considerations

The participants meet several persons during assessment, testing and treatment. Thus, to create predictability, continuity and safety, each participant is allocated a project coordinator who is responsible for their clinical pathway. Challenges and adverse events during treatment are handled by the project coordinator according to internal procedures. The project coordinator also has a closing conversation with the patient at the end of treatment to inform participants about follow-up testing and give them the opportunity to give feedback on treatment and research participation. The assessments at each test point in the study are allocated across several days to minimise strain on participants.

The development of cognitive impairment can become evident during the assessment phase. This can cause participants stress and potentially aggravate psychiatric symptoms. In cases where we document cognitive impairment during the initial assessment, the project coordinator excludes the participant from further participation. The project coordinator offers to refer the participant to gerontopsychiatric services.

If conditions that exclude patients from participating in the study are disclosed or develop during treatment, the participant will be discontinued from trial participation. In such cases, the participant will continue treatment or be referred for other suitable treatment, depending on the reason for discontinuing trial participation. If patients are in acute need of further treatment following ordinary trial termination, they are offered services as necessary at Solli DPS or other suitable facilities. All participants are insured through The Norwegian Patient Injury Compensation.

We conducted a feasibility study of four participants who received CBT + PE in March 2016 to August 2017. One of the ethical arguments for conducting the feasibility study was to ensure that the total load of testing and treatment was within the limits that the participants could tolerate. We also wanted to test the coordination of testing and treatment. Results from the feasibility study have been presented at two conferences. Further, we presented preliminary results and our experiences with the implementation of the physical exercise protocol at the 7th International Conference of Physiotherapy in Psychiatry and Mental Health in Reykjavik [101]. We presented the feasibility study and results at the 48th Annual Congress of the European Association for Behavioural and Cognitive Therapies [102]. Our conclusions from the feasibility study are that the combination of physical exercise and CBT appears to be feasible and potentially beneficial in our target population.

### Power analysis and statistical analysis

Power estimates were calculated using the G* Power 3 programme [103]. Treatment effects will be investigated by an intention-to-treat approach to data analysis using a hierarchical linear model, whereby therapists will be included at the second level to account for potential nesting by this factor. Power analysis for effects are estimated with conservative repeated measures analysis of variance (ANOVA) (within-between interactions). For the primary outcome measure, based on previous studies [22, 28], the difference in effect size is set to *d* = 0.35, alpha = 0.05, correlation between repeated measures 0.5, two measurements (pre/post), a total of 70 subjects (*n* = 35 in each group) yielding power above 0.82. Moderators and mediators will be investigated using linear multiple regression, fixed model, single coefficients. An effect size set to *f*^*2*^ = 0.15, two-tailed tests, alpha = 0.05 and *N* = 70 yields power above 0.95 with four predictors. For the subset of functional MRI (fMRI) assessments the following statistics and power analysis will be applied: task-fMRI data will be analysed using General Linear Model in SPM12 for pre-processing and single subject and group analyses, GIFT [104] to investigate the functional and structural data, and arterial spin labelling (ASL) data will be analysed in SPM12. Region of interest (ROI) will be analysed for both structural and functional data (SPM12) to extract blood oxygen level dependent (BOLD) responses to test correlation with specified variables. Expecting percent signal changes of approximately 0.5% at the single voxel and spatial smoothing at full width at half maximum (FWHM) of 5 mm (provides an estimated effect size of *d* = 0,7), using the two-tailed independent samples *t* test, an allocation rate of 1, alpha set to 0.05, correlation between repeated measures 0.5 and a total sample size of 80 (40 in each group) yields power above 0.87. For differences between pre-treatment and post treatment data using the same parameters, the matched-pairs *t* test with 20 subjects yields power above 0.90. Treatment effects are estimated using conservative repeated measures ANOVA (within-between interaction), and conservatively setting the estimated value of *d* = 0.35, alpha set to 0.05, two measurements (pre, post), correlation between repeated measures 0.5, and 60 subjects (*n* = 20 per group) yields power above 0.83. Based on these calculations, the estimated power values indicate that risk of committing type II errors is adequately controlled.

To control for type 1 errors, the secondary clinical outcome inventories will be assessed in two stages, initially together, and if significant they will be analysed separately. Pre-treatment differences on continuous and categorical demographic variables between the two treatments will be assessed using the independent samples *t* test and Fisher’s exact test, respectively. Other changes and differences in continuous variables will be assessed using the repeated and independent samples *t* test, and Fisher’s exact test and McNemar’s test to determine proportional differences between or within groups, respectively. Repeated testing within the same domain will be controlled for multiple testing using the Holm-Bonferroni method [105].

Missing data will be handled within the hierarchical linear model analyses. For analyses where this is not appropriate, last observation carried forward (LOCF) will be applied. For inventories, missing data will be replaced by the mean, provided that no more than 20% of values/item for this measure are missing. If more than 20% of values/item are missing, data will be considered invalid, and will be handled as missing as described. The potential impact of compliance with the different treatment elements will be addressed in separate analyses.

### Dissemination of results

We will submit results for publication in high impact, open access, international peer-reviewed journals. We will also communicate results through public media and give lectures to relevant user groups, such as health care practitioners in specialised health services, municipal healthcare practitioners, low-threshold preventive measures and voluntary organisations.

## Discussion

Older adults are a rapidly increasing group. GAD is frequent in this segment of the population, and older patients with GAD often do not receive adequate and effective help through the current healthcare systems, due to priorities favouring younger adults or because of the absence of proper healthcare programmes. Comorbid somatic and psychiatric illnesses are common in GAD, and create a socioeconomic burden [7]. Thus, it is also highly socioeconomically relevant to develop effective treatment for older adults. More importantly, effective treatment can lead to an increase in quality of life for the individual suffering from a psychiatric disorder, as well as improving comorbid somatic disorders. As far as we know, the knowledge on augmented treatment for older adults with GAD is scarce, although previous studies have yielded promising findings. As GAD is prevalent in older adults, and the impact of treatment for this disorder seems reduced with age, it is essential to develop more effective interventions for this growing segment of our population. To the best of our knowledge, no previous studies have investigated CBT augmented with physical exercise in older adults with GAD.

The aim of the present study is to compare the effects of CBT combined with either physical exercise or telephone call attention placebo. We expect that participants receiving CBT + PE will have greater symptom reduction assessed by the PSWQ than participants receiving CBT + AP. Further we expect physical exercise to be an efficient add-on treatment strategy for older adults with GAD because of the benefits of physical exercise on cognitive functions, mediated by changes in BDNF and HRV. We do not expect telephone calls to affect cognitive functions through these mediators, although adding telephone calls to CBT might provide a placebo effect. With the telephone calls, we try to control for the added human contact in the physical exercise condition. The added attention could increase the participants’ focus on the CBT, and as such enhance the treatment efficacy. However, we take great care not to focus on therapeutic content, and kindly ask the participant to discuss the therapy with their CBT therapist, if they bring up CBT-related themes or questions. We also instruct the physical exercise therapists to refer to the CBT therapists for CBT-related question and themes.

In addition to expecting more symptom reduction on the PSWQ in participants receiving CBT + PE, we also expect these participants to gain improved HRV and elevated baseline and acute BDNF levels at the post-treatment and follow-up measurements, as compared participants receiving CBT + AP. We will also explore whether these measures change over treatment in both treatment arms, and if so, how this change evolves over the follow-up measures. Furthermore, we expect CBT + PE to positively affect cognitive functions as measured by our neuropsychological test battery, as compared to CBT + AP. One potential limitation to this approach is the learning effects in repeated neuropsychological testing. We do expect learning effects, especially with the Wisconsin Card Sorting Test, and the California Verbal Learning Test. However, we control for this by repeating the testing in both treatment conditions and by comparing the groups.

The current study has both strengths and limitations that should be noted. One of the strengths of this study is the large number of tests and measures at multiple time points. This allows for investigation of mediators of treatment efficacy, which otherwise would be impossible to measure. However, the number of tests and measures could also pose more strain on participants, which might increase the risk of drop-out at the post-treatment and follow-up test points. We work actively to reduce this risk by coordinating the tests and treatment closely, and calling participants to schedule follow-up tests. By administering many questionnaires, we also have an increased risk of missing data. We monitor this by being physically present or available to participants as they complete the questionnaires, which allows us to reduce the number of missing responses.

With this study, we aim to fill some of the gaps in the existing knowledge on GAD in older adults. The study design allows us to investigate the effect of physical exercise as an augmentation to CBT. We also investigate long-term effects through follow up of the participants; additionally, we include several measures that allow us to examine both treatment effects and possible mediators and moderators of treatment outcome. Some important implications of this study will be to ensure accurate and more available treatment programmes for older adults.

## Trial status

The study is registered on ClinicalTrials.gov (NCT02690441). The Regional Ethical Committee has approved the study, reference number 2015/2189, approved 2015.11.15. The study opened for recruitment in September 2017, and we plan to conclude data collection in December 2020.

## Additional file

Additional file 1: SPIRIT 2013 checklist: recommended items to address in a clinical trial protocol and related documents. (DOC 121 kb)

## Abbreviations

ADIS-IV: Anxiety Disorders Interview Schedule for DSM-IV
ANOVA: Analysis of variance
BDNF: Brain-derived neurotropic factor
CBT + AP: Cognitive behaviour therapy with adjunct telephone call attention placebo
CBT + PE: Cognitive behaviour therapy with adjunct physical exercise
CBT: Cognitive behaviour therapy
CTACS: Cognitive Therapy Adherence and Competence Scale
ECG: Electrocardiogram
fMRI: Functional magnetic resonance imaging
GAD: Generalised anxiety disorder
HRV: Heart rate variability
IPAQ: International Physical Activity Questionnaire
M.I.N.I.: Mini International Neuropsychiatric Interview
MRI: Magnetic resonance imaging
PE: Physical exercise
PEXACOG: Physical exercise augmented cognitive behavior therapy for older adults with generalised anxiety disorder
PSWQ: Penn State Worry Questionnaire
WAIS-IV: Wechsler Adult Intelligence Scale - Fourth Edition
