# Correction to: Physical exercise augmented cognitive behaviour therapy for older adults with generalised anxiety disorder (PEXACOG): study protocol for a randomized controlled trial

**DOI:** 10.1186/s13063-020-04239-9

**Published:** 2020-04-06

**Authors:** Silje Haukenes Stavestrand, Kristine Sirevåg, Inger Hilde Nordhus, Trond Sjøbø, Trygve Bruun Endal, Hans M. Nordahl, Karsten Specht, Åsa Hammar, Anne Halmøy, Egil W. Martinsen, Eva Andersson, Helene Hjelmervik, Jan Mohlman, Julian F. Thayer, Anders Hovland

**Affiliations:** 1 Faculty of Psychology, University of Bergen, Box 7800, NO-5020 Bergen, Norway; 2 Solli DPS, Osvegen 15, NO-5228 Nesttun, Norway; 3 Faculty of Medicine, University of Oslo, Box 1078, Blindern, NO-0316 Oslo, Norway; 4 Department of Mental Health, Norwegian University of Science and Technology, Box 8905, NO-7491 Trondheim, Norway; 5 Faculty of Medicine, K.G. Jebsen Centre for Neuropsychiatric Disorders, University of Bergen, Box 7800, NO-5020 Bergen, Norway; 6 The Swedish School of Sport and Health Sciences, GIH, Box 5626, SE-114 86 Stockholm, Sweden; 7 Department of Psychology, William Paterson University, 300 Pompton Road, Wayne, NJ 07470 USA; 8 Department of Psychology, Ohio State University, 1835 Neil Avenue, Columbus, OH 43210 USA; 9 St.Olavs Hospital HF, Nidaros DPS, Box 3250, Sluppen, NO-7006 Trondheim, Norway; 10 Division of Mental Health and Addiction, Oslo University Hospital, Oslo, Norway; 11 Kronstad DPS/Division of Psychiatry, Haukeland University Hospital, Box 1400, NO-5021 Bergen, Norway

**Correction to: Trials (2019) 20:174**

**https://doi.org/10.1186/s13063-019-3268-9**

Following the publication of our article [1], we have become aware of one error in the exclusion criteria, inconsistencies in Figs. 1 and 2, and a typo in the reference list.

**Fig. 1:**
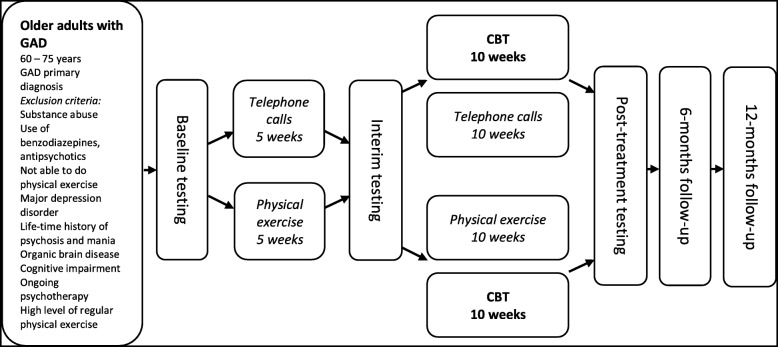
Study design. Visual presentation of the study design, including sample, assessments, and interventions. GAD, generalised anxiety disorder; CBT, cognitive behaviour therapy

**Fig. 2:**
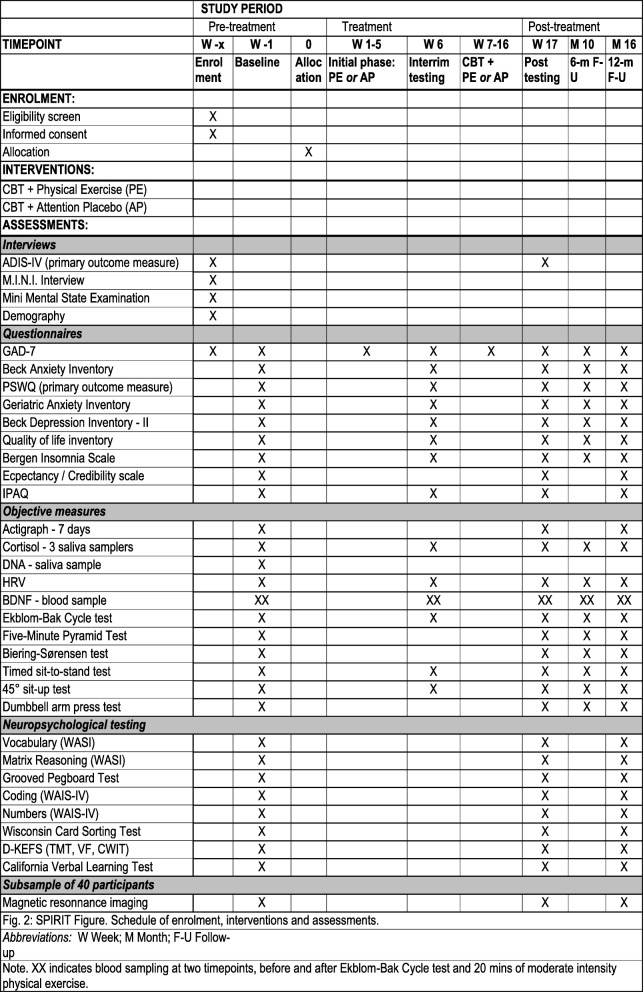
Standard protocol items: recommendation for interventional trials (SPIRIT) figure. Schedule of enrolment, interventions and assessments. Overview of the measures applied in the study. CBT, cognitive behaviour therapy; ADIS-IV, Anxiety Disorders Interview Schedule for DSM-IV; M.I.N.I., Mini International Neuropsychiatric Interview; PSWQ, Penn State Worry Questionnaire; GAD-7, Generalized Anxiety Disorder 7-item scale; IPAQ, International Physical Activity Questionnaire; HRV, heart rate variability; BDNF, brain-derived neurotropic factor; WASI, Wechsler Abbreviated Scale of Intelligence; WAIS-IV, Wechsler Adult Intelligence Scale – Fourth Edition; D-KEFS, Delis–Kaplan Executive Function System; TMT, Trail-Making Test; VF, Verbal Fluency; CWIT, Color Word Interference Test

Exclusion criterion # 10 is incorrect.

Correction:

(10) participating regularly in 60 min of moderate intensity physical exercise per week, divided across 2 or more bouts of physical exercise.

The physical exercise exclusion criteria are omitted from Fig. 1 in the original publication. The correct version of Fig. 1 is included here.

In Fig. 2, the interim testing contains three tests which are not conducted at this point of measure. These are The Five-Minute Pyramid test, The Biering-Sørensen Test, and the Dumbbell Arm Press Test. The correct version of Fig. 2 is included here.

We have discovered a typo in the reference for the Expectancy / Credibility Scale. The correct reference is:

Borkovec, T. D. and S. D. Nau (1972). “Credibility of analogue therapy rationales.” Journal of Behavior Therapy and Experimental Psychiatry 3(4): 257–260.
